# Cytomegalovirus Retinitis Screening and Treatment in Human Immunodeficiency Virus Patients in Malawi: A Feasibility Study

**DOI:** 10.1093/ofid/ofz439

**Published:** 2019-11-06

**Authors:** Paulina Ocieczek, James R Barnacle, Joe Gumulira, Sam Phiri, Tom Heller, Iwona Grabska-Liberek

**Affiliations:** 1 Department of Ophthalmology, Szpital Klinczny im. Prof. W. Orłowskiego, Warsaw, Poland; 2 Daeyang Luke Hospital, Lilongwe, Malawi; 3 Lighthouse Trust, Lilongwe, Malawi; 4 Department of Global Health, University of Washington, Seattle, USA; 5 Department of Medicine, University of North Carolina School of Medicine, Chapel Hill, North Carolina, USA; 6 Department of Public Health, College of Medicine, School of Public Health and Family Medicine, University of Malawi, Malawi

**Keywords:** CD4 count, cytomegalovirus retinitis, human immunodeficiency virus, Malawi, screening

## Abstract

**Background:**

Cytomegalovirus retinitis is a treatable cause of blindness in people with human immunodeficiency virus (HIV) typically with CD4 counts <50 cells/mm^3^. Diagnosis is with indirect fundoscopy, and treatment is with intravitreal ganciclovir injections or systemic therapy. However, diagnosis and treatment are not widely available in Malawi, which has an adult HIV prevalence estimated at 10.6%. This study aimed to establish the prevalence of cytomegalovirus retinitis among people with HIV in Malawi and the feasibility of screening.

**Methods:**

Patients with CD4 counts <200 cells/mm^3^ were examined from 2 HIV clinics in Lilongwe and the main government hospital. Data were collected on antiretroviral therapy, ocular symptoms, and visual acuity. Fundoscopy was performed to investigate for features of cytomegalovirus retinitis. Retinal photographs were reviewed by an ophthalmologist. Patients diagnosed with cytomegalovirus retinitis were offered weekly ganciclovir injections, because systemic treatment was not available.

**Results:**

Five of the 102 people with HIV screened had cytomegalovirus retinitis (4.9%). All affected patients had CD4 counts <50 cells/mm^3^ (mean, 15 cells/mm^3^; range, 3–22 cells/mm^3^). Visual acuity was unhelpful in identifying those with cytomegalovirus retinitis. Symptomatically, only blurred vision was useful. Two patients consented to treatment, 1 of which improved but relapsed after defaulting.

**Conclusions:**

Cytomegalovirus retinitis screening based on CD4 count is essential to early recognition because visual acuity and symptoms are unreliable. Cytomegalovirus retinitis is a significant yet neglected public health issue in Malawi. Oral valganciclovir is essential to reduce blindness and mortality in those diagnosed but is not yet available. Further screening and advocacy are needed.

## Teaser

Despite an estimated cytomegalovirus retinitis prevalence in people with HIV in Malawi, the logistics and acceptability of intravitreal injections and financial impact on patients hinder treatment. Further data, oral valganciclovir, and strong advocacy are needed to reduce future morbidity/mortality.

Cytomegalovirus (CMV), which causes CMV retinitis (CMVR), is a member of herpesvirus family. It is usually acquired in childhood, and in low-income countries almost universal seropositivity by adulthood has been shown [1–3]. Cytomegalovirus usually remains latent unless the host’s immune system is compromised, eg, by human immunodeficiency virus (HIV). Patients with CD4 counts <50 cells/mm^3^ are particularly at risk [4]. In the era before antiretroviral therapy (ART), approximately one third of patients with HIV would develop CMVR, which was responsible for over 90% of HIV-related blindness [5]. Since the widespread availability of ART, the prevalence of CMVR in high-income countries has significantly decreased [6]. The prevalence and impact of CMVR in sub-Saharan African countries are less well understood.

Cytomegalovirus retinitis must be considered as just one end-organ manifestation of a severe systemic disease. The association between CMV infection and mortality is longstanding. The first 5 index cases ever reported of HIV died with evidence of CMV infection [7]. In the pre-ART era in high-income countries, disseminated CMV infection was found in 38%–59% of people with HIV (PWH) at autopsy [8, 9]. Cytomegalovirus viremia is a significant predictor of death [10]. Cytomegalovirus retinitis is associated with early mortality making it particularly important to diagnose. A pre-ART study from Togo found a mean survival of 22 days after a diagnosis of CMVR [11], and recently in South Africa, 41.7% of patients with CMVR defaulted or died in the 8 months after diagnosis [12]. Identifying CMVR goes beyond the eyes; It [identifying CMVR] risk stratifies for mortality.

Early symptoms of CMVR may include scotoma, floaters, flashes, or blurred vision [13]. The gold standard for diagnosis is indirect fundoscopy with fully dilated pupils. On examination, CMVR manifests as retinal necrotizing lesions seen as yellow-white retinal infiltrates or “cotton-wool spots,” frequently associated with intraretinal hemorrhages. Lesions spread inwards from the periphery of the retina in a “brushfire pattern”. Two thirds of patients have unilateral disease at presentation [14]. If left untreated, CMVR leads to progressive necrosis of the retina, destroying it within 3 to 6 months [15]. Blindness is irreversible and is caused by direct damage to the macula or optic nerve or by retinal detachment. Visual loss may also be caused by CMV-related immune recovery uveitis (IRU), an intraocular inflammatory reaction which may occur in up to 20% of patients with pre-existing CMVR after the initiation of ART [16].

When recognized, CMVR in resource-limited settings is often treated inadequately due to poor drug availability. Besides ART, international guidelines advise systemic treatment with either oral valganciclovir or intravenous ganciclovir, foscarnet, or cidofovir [17]. Systemic treatment reduces disease in the contralateral eye, visceral disease, and mortality [18–20]. In addition, weekly intravitreal ganciclovir injections should be given in immediate sight-threatening disease for 2 to 3 weeks. In resource-limited settings, it is advised to start ART immediately despite the risk of IRU because of the high early mortality risk [21]. In patients treated with intravitreal injections alone, weekly injections are advised until active CMVR has resolved, and the patient has received both anti-CMV and ART treatment for at least 3 months, with CD4 ≥100 cells/mm^3^ or >50 cells/mm^3^ above baseline [22]. A successful approach in South Africa gave biweekly injections of 2 mg of ganciclovir in 0.08 mL for the first 2 to 3 weeks followed by weekly injections until immune reconstitution [23].

Malawi continues to have a significant burden of HIV infection. The prevalence of HIV infection among adults aged 15–64 is estimated at 10.6% corresponding to approximately 900 000 people [24]. In Lilongwe district, the prevalence is estimated at 11.5%. At diagnosis, 68.3% of PWH aged 18–64 are already immunosuppressed with a CD4 count <500 cell/mm^3^ [24].

Our study aimed to prospectively screen newly diagnosed PWH or those failing on ART for CMVR independent of existing symptoms to establish the prevalence in this patient population. In addition, ocular symptoms were recorded to establish their predictive value for CMVR in PWH. We discuss the possible benefits and obstacles of a CMVR screening programme in a low-resource setting.

## MATERIALS AND METHODS

### Setting

Lighthouse (LH) Trust is a World Health Organization (WHO) recognized Center of Excellence for integrated HIV prevention, treatment, care, and support. Lighthouse operates 2 large urban HIV clinics in Lilongwe, Malawi’s capital city. One is located at Kamuzu Central Hospital (LH-KCH), the tertiary referral hospital for the central region. The other, the Martin Preuss Center (LH-MPC), is in the downtown area of Lilongwe. On average, 93 and 353 new initiations on ART per month were recorded during 2017. The Lions Sight First Eye Hospital at KCH has 2 ophthalmology consultants, and the remaining staff are clinical officers and optometrists.

### Methods

Between December 2017 and April 2018, patients were referred to the eye clinic from 3 locations; LH-KCH, LH-MPC, and the KCH medical inpatients. CD4 counts were done routinely for all patients initiating ART or clinically failing on their ART. Patients with CD4 <200 cells/mm^3^ from LH-KCH and the KCH medical wards were referred to the eye clinic as part of their work-up. Patients from LH-MPC were only referred with CD4 <100 cells/mm^3^ to minimize logistical efforts. Inpatients were only referred if they were stable enough to be transferred to the eye clinic.

As part of the eye exam, patients were asked about eye symptoms, and visual acuity was checked using a standard Snellen Chart with and without correction for refractive error. Slit-lamp examination of the anterior segment and fundus with a fully dilated pupil was performed on each patient. Pupils were dilated using tropicamide 1% solution. Eye examinations were performed in the Lions Sight First Eye Clinic by a single trainee ophthalmologist (P.O.). The diagnosis of CMVR was based on clinical examination of the retina. A photo of each fundus was taken using a TopCon TRC NW6S retinal camera. A consultant ophthalmologist (I.G.-L.) reviewed all images for quality assurance. Basic patient data including age, sex, duration, as well as regimen of ART and CD4 count were collected. A LH clinical officer translated for those participants who did not speak English.

Patients with evidence of CMVR on fundoscopy were treated with intraocular ganciclovir. The drug was procured through LH because it is not widely available through Malawi’s public sector, but because the amount injected per individual patient is small the cost was low. The affected eye was injected with 2 mg of ganciclovir intravitreally, 4 mm from the limbus, using a 1-mL insulin syringe and 30-guage needle. All injections were performed by a trainee ophthalmologist (P.O.) with proparacaine hydrochloride 0.5% drops used for local anaesthesia. The treatment is standard treatment according to hospital practice.

The Malawi National Health Science Research Committee provides general oversight to LH clinics and granted approval (Protocol no. 829) for the routine collection and use of clinical and programmatic data of PWH for monitoring and evaluation, as was used in case of this study. Witnessed verbal consent to screening and treatment was acquired for each patient in accordance with the Declaration of Helsinki guidelines. Statistical analysis was done to assess the relationship between ocular symptoms and CMVR.

## RESULTS

One hundred and two PWH with CD4 counts <200 cells/mm^3^ were referred for screening. Fifty-seven (55.9%) of them were male; the mean age was 36 years (range, 13–69). Eighty-nine patients (87%) were on ART at the time of screening. Of those, 40 had been on medication for less than 1 month, 17 for between 1 month and 1 year, and 32 for more than 1 year. Most of the patients treated (80.9%) currently received the standard first-line ART used in Malawi consisting of tenofovir, lamivudine, and efavirenz. Sixty patients (58.8%) had a CD4 count <50 cells/mm^3^ at the time of examination, 32 (31.3%) had 50–99 cells/mm^3^, and 10 (9.8%) had 100–200 cells/mm^3^. The most common eye symptoms reported during screening were blurred vision (24 patients), itching (11), tearing (9), floaters (6), and photophobia (7). The patient characteristics are summarized in Table 1.

**Table 1.** Patient Characteristics and Ocular Symptoms

Characteristics and Symptoms	All Patients (n = 102)	CMV Retinitis Patients (n = 5)	No CMV Retinitis (n = 97)	*P* Value^a^
Characteristics				
Age (mean)	36 years	37 years	36 years	
Male sex	57 (55.8%)	5 (100%)	52 (53.6%)	
CD4 (mean)	50 cells/mm^3^	15 cells/mm^3^	51 cells/mm^3^	.0004
CD4 <50 cells/mm^3^	60 (58.8%)	5 (100%)	55 (56.7%)	
ART <1 month or untreated	53 (51.9%)	1 (20%)	52 (53.6%)	.142
Symptoms				
Blurring	24 (23.5%)	3 (60%)	21 (21.6%)	.049
Itching	11 (10.7%)	1 (25%)	10 (10.3%)	.496
Tearing	9 (8.8%)	0	9 (9.3%)	
Floaters	6 (5.8%)	1 (25%)	5 (5.2%)	.169
Photophobia	7 (6.8%)	0	7 (7.2%)	

Abbreviations: ART, antiretroviral therapy; CMV, cytomegalovirus retinitis; CMVR, CMV retinitis.

^a^Statistical comparison is between those with and without CMVR.

**Figure 1.**
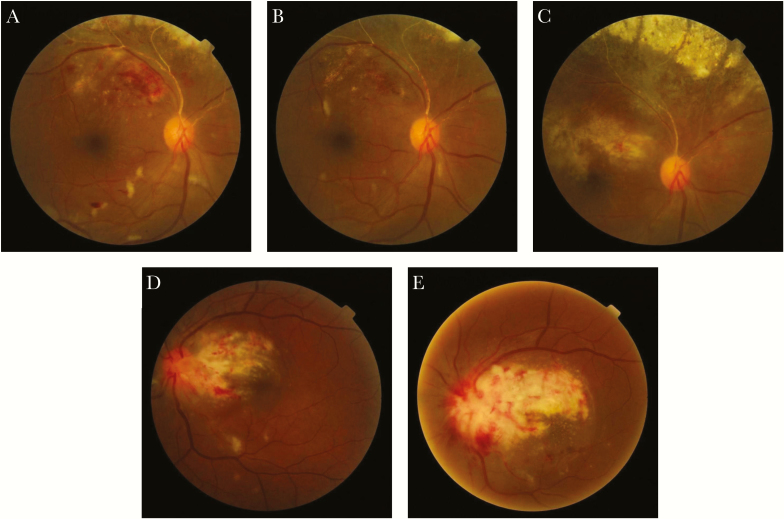
Fundus photography of patients 2 and 5. (A) Patient 2 at diagnosis. (B) Patient 2 after 6 injections. (C) Patient 2 after stopping treatment. (D) Patient 5 at diagnosis. (E) Patient 5 after 3 injections.

Typical fundoscopic findings of CMVR were found in 5 of 102 patients (4.9%) (Figure 1). Signs of HIV retinopathy were also seen in 8 of 102 patients (7.8%), 1 of whom had concomitant CMVR. All patients diagnosed with CMVR were male; the mean age was 37 years (range, 30–49). All 5 patients had CD4 counts <50 cells/mm^3^ (range, 3–22 cells/mm^3^). The mean CD4 count was 15 cells/mm^3^, significantly lower than in patients without CMVR (*P* = .0004). Three patients with CMVR complained of blurred vision, 1 complained of of itching, 1 complained of headaches, and 2 reported floaters. Visual acuity in patients diagnosed with CMVR ranged from recognizing hand movements to 6/6 in the affected eye (Table 2). Visual acuity in 1 patient (20%) was normal in both eyes. One patient was not on ART, 1 patient had been taking ART for just over 3 months, and 3 patients had been taking ART for over 2 years, although clearly with adherence or resistance problems.

**Table 2.** Visual Acuity in Patients Diagnosed With CMVR^a^

Patient	VA RE	VA LE	ART Duration (Days)	CMVR Treatment
1	**6/18**	6/9	737	No
2	**6/9**	6/6	96	Yes
3	**6/12**	6/9	1465	No
4	6/6	**6/6**	842	No
5	6/6	**HM**	NA	Yes

Abbreviations: ART, antiretroviral therapy; CMVR, cytomegalovirus retinitis; HM, hand movements; LE, left eye; NA, not applicable; RE, right eye; VA, visual acuity.

^a^Affected eye highlighted in bold.

Treatment was offered to all patients; however, just 2 patients consented to injections (Patients 2 and 5). Patient 2 received 6 ganciclovir injections within 6 weeks and substantially improved (Figure 1A before treatment, Figure 1B after treatment). Unfortunately, after 6 injections, the patient stopped attending the clinic because he could not afford the transportation costs. However, he returned after 4 weeks with significantly worse vision in the affected eye (6/24). Fundoscopy showed new scarring near the macula (Figure 1C), and his CD4 count had fallen further. The patient received 2 further injections without improvement and was lost to follow up.

Patient 5 received 4 injections without visual improvement because his macula and optic disc were already affected at diagnosis (Figure 1D before treatment, and Figure 1E after treatment). The patient tried to purchase systemic ganciclovir to prevent CMV from attacking the other eye, but systemic treatment was not available even privately in Malawi at the time. He was followed up every 2 weeks to control the other eye and was taking ART under LH supervision.

## Discussion

For a successful screening program, the disease must be prevalent enough to make screening worthwhile, and the method of screening must be sensitive, feasible, inexpensive, and acceptable to the population. In addition, if the screening is positive, there must be a robust referral system and an effective, available, and accessible treatment. Our discussion will examine these criteria in the context of CMVR screening in Malawi and in other low-resource settings.

### Disease Prevalence

Our screening program demonstrated a prevalence of 4.9% in patients with CD4 counts <200 cells/mm^3^, which increased to 8.3% when only patients with CD4 counts <50 cells/mm^3^ were selected. The lower cutoff for patients from LH-MPC meant a low number (9.8%) of patients with CD4 100–200 cells/mm^3^, which may have exaggerated the prevalence in patients with CD4 counts <200 cells/mm^3^. The prevalence for patients with CD4 counts <50 cells/mm^3^ is more useful. One possible source of underreporting in this study is the screening of stable inpatients only. Those patients unstable with other opportunistic infections were more likely to have CMVR but were not included in our screening. A long duration between diagnosis of HIV and screening may also underestimate the true prevalence if the most unwell patients died before being screened. Although there are no data showing the duration between diagnosis of HIV and screening, the median number of days on ART at screening was 41. Patients at LH are offered ART on their day of diagnosis, so it is reasonable to suggest that most patients had been diagnosed within the past 2 months when screened.

Several studies have suggested that the prevalence of CMVR is lower in Africa than Asia [25]. From small cohorts of patients with CD4 counts <50 cells/mm^3^, prevalence of CMVR of 2% in South Africa and 8% in Uganda were previously reported [26]. Data about the prevalence of CMVR in Malawi are limited. One study in Blantyre found just 1 case of CMVR out of 191 WHO stage 3 or 4 PWH presenting to hospital with fever (0.5%) [27]. Beyond this, there have been no studies examining CMVR and the role for screening in Malawi.

All CMVR patients had CD4 counts <50 cells/mm^3^, consistent with the fact that most CMVR occurs in patients with advanced HIV [4]. All of the CMVR patients were relatively young and economically active; visual loss is a big social and economic problem. Prevalence and impact justify targeted screening in our patient population.

### Screening

Fundoscopy is an excellent screening tool for CMVR. It is sensitive, even in the hands of a clinician without specific training in ophthalmology. A screening program in Myanmar demonstrated that HIV clinicians with no background in ophthalmology can be effectively trained to screen and treat CMVR with a 4-day workshop delivered by a consultant ophthalmologist [28]. It is quick, noninvasive, inexpensive, acceptable to patients, and gives immediate results. Nevertheless, it does have limitations. Although clinicians can be trained in its use quickly, it still requires training from an ophthalmologist, with a system of quality assurance. Fundoscopes are expensive and require a power source, and dilating eye drops such as tropicamide 1% must be available. For rural or outreach screening, these may be real obstacles.

Untargeted screening would be overwhelming with almost 1 million PWH in Malawi. Screening must be targeted based on CD4 count or symptoms. Ocular symptoms and visual acuity were not useful predictors of CMVR. Of the 102 patients screened, just 43 (42%) and 40 (39%) had normal vision in their right and left eyes, respectively. Of those with CMVR, vision ranged from normal to recognizing hand movements only. Blurred vision was reported in 3 of 5 (60%) and was significantly associated with CMVR (*P* = .049), but more than one fifth of participants without CMVR also complained of it. Other symptoms asked about were unhelpful in predicting CMVR. Although a study screening PWH in Thailand also concluded that eye symptoms and impaired visual acuity were poor diagnostic indicators for CMVR [29], a screening program in the United States found that PWH with new ocular symptoms were much more likely to have CMVR, with visual field defects and flashes particularly useful indicators [30]. There are 2 possible explanations. First, the reliability of our symptom reporting was limited by cultural and language barriers. Often participants would admit to certain symptoms only when asked specifically. The interpretation of “blurred vision” can be different to different people and when translated, further opportunity for misinterpretation may be added. A script was not used for translating; the exact wording was left to the translator’s discretion. Second, the prevalence of other untreated eye problems in Malawi such as refractive disorders or other retinopathies is likely to be higher. These may be present for years, so that the patient no longer thinks of them as symptoms, which could mask features of CMVR.

When considering future screening programs, even if symptoms were a good predictor of disease, it would not be a reliable way to identify those at risk of CMVR. Patients in Malawi typically do not seek medical attention for eye symptoms before sight is significantly impaired. The decision to seek care is, among other things, influenced by educational level, stigma, knowledge of existing services, previous experiences. and perceived costs. Once a decision to access ophthalmology services has been made, they are often inaccessible, even within the same city, due to transportation costs, opportunity costs from missing work, treatment costs, and overt or covert extra costs at the facility. Waiting for PWH to present with ocular symptoms catches them too late, and misses those without symptoms.

Narrowing down screening to only those patients with low CD4 counts is more appealing. All 5 of our CMVR patients had CD4 counts <50 cells/mm^3^. We used a cutoff <200 cells/mm^3^, which was probably higher than necessary; a screening program in Myanmar used a cutoff <100 cells/mm^3^. They found a median CD4 count in those diagnosed with CMVR consistently <50 cells/mm^3^ but a 75th percentile as high as 87 cells/mm^3^, implying that a cutoff of <50 cells/mm^3^ for screening may be inadequate [28]. Cases occurring in patients with CD4 >100 cells/mm^3^ appear only in case reports [31, 32]. However, screening those with CD4 <50 cells/mm^3^ in Malawi seems reasonable based on our data and in line with previous screening programs in sub-Saharan Africa [26].

The process of referring patients with low CD4 counts for screening is important. In our case, HIV clinicians were asked to contact the eye clinic directly if they had a result for one of their patients <200 cells/mm^3^. The eye unit was next to the HIV clinic making it an easy journey. Most of our patients were screened on the same day as identifying a low CD4 count, and almost all within 1 week. If the patient must return to a different place on a different day, then the attrition would increase enormously. Ideally, screening would take place inside the HIV clinic with a pathway flagging low CD4 counts and directing patients for same-day screening.

Finally, a screening program based on CD4 counts will only work if patients are having their CD4 counts tested. Therefore, diagnosis of CMVR relies not only on the diagnosis of HIV, but also the testing of CD4 count that has become less frequent now that PWH are started on ART regardless of disease progression. Many CD4 machines in Malawi have been decommissioned with the advent of the new WHO guidelines, which would hinder CD4-based screening.

### Treatment

Once CMVR has been diagnosed, weekly antiviral injections for a minimum of 3 months is a logistical and financial challenge for patients and staff. Each vial of ganciclovir costs US $7 and could be used for multiple injections, making it very cost effective. Vials of reconstituted ganciclovir can be stored safely at room temperature for up to 35 days [33]. To prevent CMVR recurrence, regular injections are crucial until the CD4 count is consistently ≥100 cells/mm^3^. Contralateral spread cannot be prevented, but ART and regular follow up can reduce the risk and identify new retinal changes early. Just 2 of 5 patients diagnosed with CMVR agreed to weekly intravitreal injections, and just 1 improved clinically. Even in that case, after the sixth injection, the patient stopped attending until deterioration again in his affected eye prompted him to return. It is clear that there are significant patient barriers to attending for treatment. The main barrier for all 5 patients was the transportation costs, which can be overwhelming. Despite knowing the implications of stopping treatment, these patients could not regularly attend the hospital. Undoubtedly, patient education on CMVR and its complications is essential to maximizing treatment uptake.

Valganciclovir, the prodrug of ganciclovir, has excellent oral bioavailability and has been shown noninferior to intravenous ganciclovir for treating CMVR [34]. It requires 2 to 3 weeks’ induction of 900 mg twice daily, after which 900 mg once daily is given as maintenance. Valganciclovir is on the WHO Model List of Essential Medicines for treatment of CMVR, yet despite generics being available, the price remains prohibitively high in Malawi and access is very limited. If the price were to fall considerably, it would revolutionize the treatment of CMVR, which could be managed in a community setting.

The lack of WHO guidelines for screening, diagnosis, and treatment contributes to the ongoing neglect of CMVR. Cytomegalovirus retinitis is not included in the WHO and International Agency for the Prevention of Blindness (IAPB) program VISION 2020 that, by the year 2020, aims to eliminate avoidable blindness [16]. East and Southern Africa suffer particularly from a lack of data on CMVR; at the time of writing, Mozambique, Zambia, Zimbabwe, Kenya, and Lesotho do not even have reported cases in the literature. With so few cases diagnosed, there is little incentive to prioritize CMVR or to provide valganciclovir treatment. The problem has been confronted in South East Asia, particularly in Myanmar where systematic screening, research publications, and advocacy have raised awareness and seen valganciclovir become available in the public sector. Although treatment is still expensive, it is provided by non-governmental organizations (NGOs); Medécins sans Frontières has been providing treatment there since 2014.

## Conclusions

Screening for CMVR in Malawi faces several challenges. Given the severity of the disease, its prevalence makes it an important health issue for patients living with HIV. Ocular symptoms are unhelpful, and screening should be focused on all patients with CD4 counts <50 cells/mm^3^. As one of our cases shows, intraocular injections can be an effective treatment for local disease with good adherence when combined with ART, but it is poorly accepted by patients, especially if asymptomatic.

Beyond Lilongwe, a national program must be accessible geographically and financially for PWH, which means having CMVR screening and treatment set up at HIV clinics across the country equipped with trained staff. These clinics need access to CD4 analyzers both for identifying those in need of screening and to determine when treatment can be stopped.

Robust data in sub-Saharan Africa are required to draw the attention of governments, donors, and NGOs. With recognition comes funding, as well as pressure to reduce drug prices. This paper adds substantially to a limited body of data in the region by demonstrating that CMVR is a public health issue and can be screened for effectively. It should be remembered that CMV is a systemic disease associated with significant mortality, and the availability of valganciclovir is essential, not only for saving sight, but also lives.
